# Epidemiology of Immune-Mediated Glomerulopathies before and after SARS-CoV-2 Vaccination: A Tertiary Referral Hospital Experience

**DOI:** 10.3390/jcm12062420

**Published:** 2023-03-21

**Authors:** Jorge Iván Zamora, Marina López-Martínez, Marc Patricio Liebana, Juan Carlos Leon Román, Sheila Bermejo, Ander Vergara, Irene Agraz, Natalia Ramos Terrades, Maria Antonieta Azancot, Nestor Toapanta, Maria Alejandra Gabaldon, Maria José Soler

**Affiliations:** 1 Department of Nephrology, Vall d’Hebron University Hospital, 08035 Barcelona, Spain; 2 Centro de Referencia en Enfermedad Glomerular Compleja del Sistema Nacional de Salud (CSUR), Vall d’Hebron University Hospital, 08036 Barcelona, Spain; 3 Department of Pathology, Vall d’Hebron University Hospital, 08035 Barcelona, Spain

**Keywords:** immune-mediated glomerulopathy, flare, vaccine, SARS-CoV-2

## Abstract

Background: Vaccination is a known trigger for the appearance of immune-mediated glomerulopathies (IMG). The appearance of IMG after SARS-CoV-2 vaccination with suspected causality has been described. Our aim is to analyze the incidence of IMG flares before and after SARS-CoV-2 vaccination in our center. Methods: All persons with native kidney biopsy (KB) from January 2019 to March 2022 in our center were included in the study. We compared the incidence of IMG before and after the start of vaccination. We also collected information about whether the patients had received a SARS-CoV-2 vaccine or have suffered from COVID in the six weeks before the IMG. We also evaluated the analytical characteristics of the outbreaks. Results: A total of 386 KB were studied. Of them, 86/218 (39.4%) were IMG performed pre- and 85/168 (50.6%) post-SV (029). The incidence of idiopathic nephrotic syndrome (INS), studied separately, was also significantly increased post-vaccination (*n* = 18 (10.7%)) compared to pre-vaccination (*n* = 11 (5%)) (*p* = 0.036). There were no differences in the incidence of vasculitis or IgA nephropathy. Up to 17 (20%) flares occurred 6 weeks before SARS-CoV-2 vaccination and only 2 (2.4%) within the first 6 weeks after SARS-CoV-2 infection. Within those 17 flares, the most common diagnosis was IgAN (*n* = 5 (29.4%)); a total of 14 (82.4%) received an mRNA vaccine and 9 (52.9%) took place after the 1st vaccine dose. There were 13 cases of minimal change disease (MCD) with debut/recurrence pre-SV and 20 MCD with debut/recurrence post-SV (*p* = 0.002). Conclusions: The incidence of IMG, INS and MCD flares in our center increased significantly after SARS-CoV-2 vaccination. Importantly, 20% of IMG flares took place within the first 6 weeks after receiving a vaccine dose, with the first dose being the riskiest one and IgAN the most frequent diagnosis.

## 1. Introduction

Global vaccination against SARS-CoV-2 has prevented an estimated of 14.4 million deaths, representing a global reduction in mortality of 79% in the first year after vaccination [1]. However, side effects related to the SARS-CoV-2 vaccine questioned its security in some cases. Although a minority of subjects have been affected, immune-mediated disorders such as thrombosis with thrombocytopenia [2], myocarditis [3], Guillain–Barré syndrome [4] and glomerulonephritis [5,6] after COVID-19 vaccination have been described. Currently, the risk of immune-mediated disorders as a consequence of vaccination is still a controversial and debated issue.

De novo or relapsing glomerular diseases such as minimal change disease (MCD), idiopathic nephrotic syndrome (INS) and IgA nephropathy (IgAN) have also been described after pneumococcal, influenza and hepatitis B vaccines [7,8]. It is believed that immunological phenomena associated with vaccination are produced by a mechanism called “molecular mimicry”. This mechanism may be caused by the homology between the vaccine and human antigens, in which the similarities between a foreign and a self-antigen favor the activation of autoreactive B or T cells [9,10]. In addition, the SARS-CoV-2 infection itself has also been identified as a trigger of immune-mediated diseases [11,12]. Interestingly, it has been postulated that, in contrast with live virus vaccines and natural infection, mRNA vaccines produce higher antibody titers and more potent CD4+, CD8+, and T follicular helper B cell (TFH) responses [13].

In the first months after the start of mass COVID-19 vaccination, Lebedev L. et al. published a case of a 50-year-old man who developed acute renal failure in the context of MCD four days following a SARS-CoV-2 vaccination [14]. Subsequently, there have been multiple case series of patients who developed de novo glomerulonephritis or relapses following vaccination against SARS-CoV-2 [15,16,17,18,18,19,20,21,22,23,24,25]. The timing which has been established could indicate causality reaching up to 6 weeks after vaccination [6,26] and IgAN and MCD seem to be the most frequent glomerulopathies observed [16]. It has been theorized that RNA-based vaccines could induce IgA nephropathy by stimulating the Peyer’s patches to produce galactose-deficient IgA1 [27]. Regarding MCD, it is known that TFH cells contribute to the development of podocyte injury, and vaccination against SARS-CoV-2 stimulates the TFH response [27].

However, it has hardly been evaluated if there is an increase in the incidence of immune-mediated glomerulopathies (IMG) after COVID-19 vaccination. Our aim is to study the possible effect of vaccination against SARS-CoV-2 on the onset and recurrence of IMG. For that purpose, we analyzed the incidence of IMG in our center before and after the start of SARS-CoV-2 vaccination in Spain.

## 2. Materials and Methods

### 2.1. Study Design and Patients

We conducted a single center, retrospective cohort study at Vall d’Hebron Barcelona Hospital Campus. All patients with kidney biopsy (KB) that had a result of an IMG from January 2019 to March 2022 in our center were included in the study. Histologic findings of focal and segmental glomerulosclerosis (GSFS), membranous nephropathy (MN), MCD, IgAN, lupus nephropathy, membranoproliferative glomerulonephritis (MPGN), C3 glomerulonephritis, fibrillary glomerulonephritis (FGN), vasculitis, anti-glomerular basement membrane disease (anti-GBM), C1q nephropathy, IgG4 disease, immunoglobulin M nephropathy (IgMN), cryoglobulinaemia, postinfectious GN and sarcoidosis were all considered as IMG (Table 1). Furthermore, INS, which included MCD and primary GSFS, was studied as a subgroup (Table 1). Patients with a previous diagnosis of MCD who presented at least one recurrence outbreak during the same period of time were also included in the study (The Ethical Committee of Vall d’Hebron University Hospital approved the study protocol (AG) 252/2018).

### 2.2. Study Follow up and Data Collection

For all patients included in the study, age, gender, basal creatinine, KB indication, laboratory test results at the time of the biopsy (creatinine, urine protein/creatinine ratio, plasma albumin, hematuria) and time to remission after treatment were recorded. A relapse of MCD was considered when nephrotic syndrome was diagnosed after a period of complete remission.

In each patient with a biopsy result consistent with an IMG or a MCD recurrence after 27 December 2020, we also assessed the history of SARS-CoV-2 infection, their vaccination status, the type of vaccine received (whether mRNA vaccine or not) and determined the time-lapse from vaccine to diagnosis of IMG (less than six weeks or more than six weeks).

### 2.3. Statistical Analysis

Data were first tested for normal distribution using the Kolmogorov–Smirnoff test. Variables with normal distribution were expressed as mean ± SD. Nonparametric variables such as proteinuria were expressed as median (interquartile range—IQR-: 25th–75th percentile) and categorical variables as percentages. An evaluation of the incidence of IMG before and after the start of vaccination (27 December 2020) and more or less than 6 weeks from the last vaccination/SARS-CoV-2 infection, as well as response to treatment, was assessed using chi-squared test. The group differences (before and after vaccination) were assessed using a sample *t*-test or Mann–Whitney U test, according to the distribution of the variables. *p* values less than 0.05 were considered statistically significant. Statistical analyses were performed using the SPSS program (SPSS version 20, Chicago, IL, USA).

## 3. Results

### 3.1. Study Population and IMG before/after SARS-CoV-2 Vaccination

A total of 386 patients had a KB in our center between 1 January 2019 and 31 March 2022, with 218 (56.4%) before the start of general SARS-CoV-2 vaccination in Spain and 168 (43.5%) after the start of SARS-CoV-2 vaccination in Spain. A total of 171 (44.3%) had a diagnosis of IMG (Table 1). Within the IMG group who were biopsied after general vaccination, there were 45 males (52.3%), with a medium age of 50 ± 18.3 years (Table 2). The most common reason for kidney biopsy in the group studied after the start of vaccination in Spain was acute renal failure or chronic kidney disease with acute exacerbation (*n* = 31 (36.5%)), with a peak creatinine level of 3.09 mg/dL (2.01–4.97) (Table 2). Laboratory characteristics were serum albumin of 3.5 g/dL (2.9–3.9) and proteinuria of 1.96 g/g (0.56–5.56) as well as 45 (52.9%) individuals presenting with hematuria (Table 2). In the group which had a diagnosis of IMG before a SARS-CoV-2 vaccination, there were 38 males (44.2%), with a mean age of 51 ± 19.4 years with the following laboratory characteristics: serum albumin of 3.5 g/dL, peak creatinine of 2.22 mg/dL (1.71–4.70), proteinuria of 1.9 g/g (0.94–6.31) and 56 (65%) individuals presenting with hematuria, with no statistical differences.

### 3.2. Incidence of IMG before and after the Start of Vaccination

**Figure 1:**
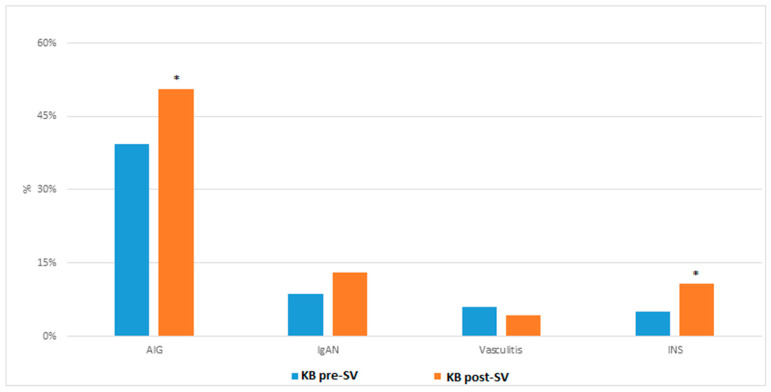
Incidence of IMG before and after vaccination. KB: kidney biopsy, SV: start of vaccination, IMG: immune-mediated glomerulopathies, IgAN: IgA nephropathy, INS: idiopathic nephrotic syndrome. * *p* < 0.05 is considered statistically significant.

The incidence of IMG before the start of vaccination (*n* = 85 (50.6%)) was significantly higher than the incidence of IMG after the start of SARS-CoV-2 vaccination (86 (39.4%)) (*p* = 0.029) (Table 3, Figure 1). The incidence of IgAN, INS and vasculitis were independently studied. Patients with biopsies with INS after the start of vaccination (18 (10.7%)) were significantly more frequent than patients with INS before the start of vaccination (11 (5.0%)) (*p* = 0.036) (Table 3, Figure 1). However, the incidences of IgAN and vasculitis before general vaccination (19 (8.7%) and 13 (6%), respectively) and the incidences of IgAN and vasculitis after general vaccination (22 (13.1%) and 7 (4.2%), respectively) were not statistically different, *p* = 0.185 and *p* = 0.430, respectively (Table 3).

### 3.3. Flares of MCD before and after Vaccination

There were a total of 33 flares of MCD in the period of study. Up to 13 (39.39%) presented a flare before the start of general vaccination in Spain and 20 (60.61%) after it (*p* = 0.002) (Table 4, Figure 1). None of these patients had a previous COVID-19 infection. There were six males (46.15%) before national vaccination and six males (30%) after national vaccination, *p* = 0.346 (Table 4). The mean age before vaccination was 44.23 ± 20.77 years and 49.50 ± 23.25 years after vaccination, with *p* = 0.512 (Table 4). The median creatinine and proteinuria before and after national vaccination were 0.65 (0.55–1.74) mg/dL and 8.67 (1.67–12.31) g/g and 0.88 (0.67–1.01) mg/dL and 4.85 (2.50–10.79) g/g, respectively (*p* = 0.302 and *p* = 0.478) (Table 4).

A total of seven (35%) of the flares which took place after general vaccination achieved remission before 1 month, while two (14.38%) of the flares which took place before general vaccination achieved remission before 1 month of treatment, with *p* = 0.005 (Table 4). There were eight remissions (61.54%) between 1–3 months after treatment in the group that had a flare before general vaccination versus eight remissions (40%) in the group that had a flare after general vaccination, with *p* = 0.02 (Table 4). Up to three patients (15%) of the group that had a flare after general vaccination went into remission between the third and sixth month, while one patient (7.69%) of the group that had a flare before general vaccination went into remission between that period of time, with *p* = 0.02 (Table 4).

Information regarding IMG diagnosis 6 weeks after SARS-CoV-2 infection or SARS-CoV-2 vaccination is shown in Table 5. Up to 17 (20%) of the 85 patients who developed an IMG after the start of vaccination were diagnosed within the first 6 weeks after receiving the vaccine. Within this group, 14 (82.4%) received an mRNA vaccine. The biopsy diagnoses were one MCD (5.9%), one FSGS (5.9%), five IgAN (29.4%), two vasculitis (11.8%), two anti-GBM disease (11.8%), three lupus nephritis (17.5%), one IgG4 disease (5.9%) and one MPGN (5.9%). There were eight (47.10%) males, with a mean age of 50.47 ± 15.88, albumin of 3.5 g/dL (3.05–4.30), creatinine of 1.75 mg/dL (0.77–5.22), proteinuria of 2.41 g/g (0.82–6.06) as well as nine (52.9%) presenting with hematuria. Up to 14 (82.4%) received an mRNA vaccine. Most of the flares (9–52.9%) took place after the first vaccine dose, with four (23.5%) coming after the second vaccine dose and another four (23.5%) after the third vaccine dose. Two (2.4%) individuals were diagnosed with IMG within the first 6 weeks after SARS-CoV-2 infection.

## 4. Discussion

Our study demonstrated that there have been significantly more IMG in our center after SARS-CoV-2 vaccination. Up to 50.6% of kidney biopsies performed in our center after SARS-CoV-2 vaccination were diagnosed with IMG, while only 39.4% of kidney biopsies performed in our center before SARS-CoV-2 vaccination were diagnosed with IMG (*p* = 0.029) (Table 3). There are scarce results in the literature about the assessment of the increase in the incidence of IMG after vaccination. Diebold et al. [28] studied the incidence of four IMG (IgAN, MCD, membranous nephropathy and necrotizing extracapillary glomerulonephritis) in the adult Swiss population during the first period of the vaccination campaign (January to August 2021), and compared it with the expected incidence based on the previous 5 years (2015–2019), but no increase in the incidence of any IMG was found. Similarly, Caza et al. [25] did not find differences between the incidence of glomerular diseases after vaccination against SARS-CoV-2 and the incidence of glomerular diseases in the 2 years prior to the COVID-19 pandemic.

Interestingly, we also found a statistically significant increase in the incidence of INS in our center after the start of vaccination (from 11 (5.0%) to 18 (10.7%)), with *p* = 0.036 (Table 3). Moreover, there were also significantly more flares of MCD after vaccination (from 13 to 20), *p* = 0.002, although with no statistically significant differences regarding gender, age, creatinine, albumin or proteinuria (Table 4). A recent publication [29] also described an increased incidence of MCD and GSFS with tip lesion after SARS-CoV-2 vaccination compared with the incidence 5 years prior to the start of vaccination. Indeed, MCD has been one of the most frequent glomerular diseases associated with SARS-CoV-2 vaccination [6,7]. It has been suggested that there is a role for cellular immunity in the development of MCD following vaccination, as mRNA vaccines trigger Tfh responses and the pathogenesis of MCD is explained by a dysregulated T-cell activation and secondary podocyte injury [27]. SARS-CoV-2 and mRNA vaccines have been associated with endothelial injury. Interestingly, recent studies have shown alterations in the glomerular endothelium in patients with MCD, suggesting that MCD associated with SARS-CoV-2 vaccination may in part be ascribed to glomerular and systemic endothelial disfunction [30,31,32]. It is worth noting that the response to the standard therapy for MCD has been suggested to be worse when it occurs around the same time as COVID-19 vaccination [6]. In contrast, in our study there was a better response to treatment in the first month of relapse in MCD flares after national vaccination than before it (Table 4).

The most frequent glomerular disease reported in the literature after SARS-CoV-2 vaccination is IgAN [5,17,19,24,25]. This effect could be in part explained by an IgA immune response against the SARS-CoV-2 spike protein, which could trigger IgA antibodies, similar to what has been already reported in influenza vaccines [17]. Moreover, some “debuts” were probably subclinical IgAN with sudden clinical manifestations after COVID-19 vaccination. By contrast, we did not find an increased incidence of IgAN before and after COVID vaccination in our cohort. However, the most frequent diagnosis of IMG flares in our center that occurred within 6 weeks of a vaccine dose was IgAN, affecting almost the 30% of the cases. The latest group analyzed separately was vasculitis, where we did not find any difference. In the group of 17 IMG flares which took place within 6 weeks of a vaccine dose, vasculitis was the diagnosis for two of them (11.8%). A systematic review written by Wu et al. [7] identified vasculitis as the third most common histopathology reported following SARS-CoV-2 vaccination, although the pathophysiology remains obscure.

Although it is almost impossible to certainly identify a causal relationship between SARS-CoV-2 vaccination and new-onset nephropathies, there is an increasing number of case reports in the literature relating glomerular diseases flares with vaccine administration [5,8,14,15,16]. The risk of a glomerular disease flare has been described from a few hours to 6 weeks after SARS-CoV-2 vaccination [6,29,33]. In accordance with this suggested link, our study observed that 17 (20%) of the IMG took place during the first 6 weeks after vaccination against SARS-CoV-2 (Table 5). In the case of clinical manifestations in the first six weeks from vaccination, such as edema or macroscopic hematuria, we recommend performing a urine test to rollout a glomerular flare. Interestingly, most of the patients (82.4%) received an mRNA vaccine and the 52.9% of cases took place after the first dose (Table 5). Independently of the real causality, the amount of reported and unreported cases described as vaccine-linked is much smaller than the cases which received the vaccine safely [34,35,36,37,38,39]. Therefore, we encourage and recommend proceeding with COVID-19 vaccination. A summary of COVID-19 vaccination recommendations was published in 2021 [17], in which patients with transplant organs, end-stage renal disease, cardiovascular diseases and immune-mediated disorders, among others, were strongly recommended to receive the SARS-CoV-2 vaccination. In these cases, only patients under treatment with prednisone above 20 mg daily, in which tapering doses is not possible, or at least within the first 6 months after anti-CD20 therapy, are situations where delaying vaccination to achieve an optimal antibody response could be reasonable [17]. Undoubtedly, when an IMG following COVID-19 vaccination has already happened, we recommend individualization of the therapeutic attitude depending on the severity of the clinical and histological manifestation of the immune-mediated flare.

Our study has limitations and strengths. It is a retrospective study and causality cannot be demonstrated. Comparing the incidence of IMG with the incidence immediately prior to vaccination initiation could be subject to bias due to confounding factors, since in the period immediately prior to vaccination, isolation measures were stricter, which also reduced exposure to agents known to cause IMG. We only have data from a single center, so we do not know if the results obtained in this study could be applicable to other populations. Finally, the lack of studies that compare the incidence of IMG before and after the start of vaccination makes it difficult to interpret results. However, it is one of the first studies to evaluate the change in the incidence of IMG after the start of vaccination. For the population studied, it confirms previous findings that were suggested in multiple reports and provides additional evidence that IMGs have increased since vaccination began. It could be used as a basis to assess the effect of SARS-CoV-2 vaccinations on IMG in other populations and on other immune-mediated diseases.

In conclusion, the incidence of IMG, INS and MCD flares in our center increased significantly after vaccination against SARS-CoV-2. Importantly, 20% of IMG flares took place within the first 6 weeks after receiving a vaccine dose, with the first dose being the most risky one and IgAN the most frequent diagnosis. However, vaccination risks are still much less probable than the consequences of coronavirus disease, thus we believe that global vaccination against SARS-CoV-2 should be still mandatory. Although causality remains unclear, further research should be performed to assess the real impact of vaccination in the IMG and its implications in the evolution and treatment of patients.

## Figures and Tables

**Table 1:** Immune-mediated glomerulonephritis (IMG) cases included in the study.

IMG (%)	171 (100)
INS (%)	29 (16.95)
MN (%)	18 (10.52)
IgAN (%)	41 (23.97)
Lupus Nephropathy (%)	31 (18.12)
MPGN (%)	11 (6.43)
C3G (%)	1 (0.5)
FGN (%)	4 (2.33)
Vasculitis (%)	20 (11.69)
Anti-GBM disease (%)	5 (2.92)
C1q GN (%)	1 (0.58)
IgG4 disease (%)	2 (1.16)
IgMN (%)	1 (0.58)
Cryoglobulinemia (%)	2 (1.16)
Postinfectious GN (%)	3 (1.75)
Sarcoidosis (%)	2 (1.16)

INS: idiopathic nephrotic syndrome. MN: membranous nephropathy. IgAN: immunoglobulin A nephropathy. MPGN: membranoproliferative glomerulonephritis. C3G: complement 3 glomerulopathy. FGN: fibrillar glomerulonephritis. Anti-GBM disease: anti-glomerular basement membrane disease. C1q GN: complement 1q glomerulonephritis. IgG4 disease: immunoglobulin G4-related disease. IgMN: immunoglobulin M nephropathy. Postinfectious GN: postinfectious glomerulonephritis.

**Table 2:** Demographic and laboratory characteristics of patients diagnosed with IMG before and after the start of SARS-CoV-2 vaccination.

	IMG before SARS-CoV-2 Vaccination	IMG after SARS-CoV-2 Vaccination	*p*
*n* (%)	86 (39.4)	85 (50.6)	***p* = 0.029**
Gender (male %)	38 (44.2)	45 (52.3)	*p* = 0.321
Age (years)	51 ± 19.4	50 ± 18.3	*p* = 0.842
Albumin (g/dL)	3.5 (2.7–4.0)	3.5 (2.9–3.9)	*p* = 0.953
Creatinine (mg/dL)	2.22 (1.71–4.70)	3.09 (2.01–4.97)	*p* = 0.180
Proteinuria (g/g)	1.90 (0.94–6.31)	1.96 (0.56–5.56)	*p* = 0.478
Hematuria (yes %)	56 (65.1)	45 (52.9)	*p* = 0.162
AKI/CKD with acute exacerbation (%)	38 (44.2)	31 (36.5)	*p* = 0.076

Data are expressed as *n* (%), mean ± SD or median (interquartile range—IQR-: 25th–75th percentiles). AKI: acute kidney injury. CKD: chronic kidney disease. *p* values less than 0.05 are considered statistically significant (bold values).

**Table 3:** Incidence of IMG, IgAN, vasculitis and INS before and after the start of vaccination.

	Pre-Vaccine	Post-Vaccine	*p*
IMG	86 (39.4%)	85 (50.6%)	***p* = 0.029**
IgAN	19 (8.7%)	22 (13.1%)	*p* = 0.185
Vasculitis	13 (6.0%)	7 (4.2%)	*p* = 0.430
INS	11 (5.0%)	18 (10.7%)	***p* = 0.036**

Data are expressed as *n* (%). IMG: immune-mediated glomerulopathies, IgAN: IgA nephropathy, INS: idiopathic nephrotic syndrome. *p* values less than 0.05 are considered statistically significant (bold values).

**Table 4:** Demographic and laboratory characteristics of patients with MCD flares before and after the start of vaccination.

	Flare Pre-Vaccine	Flare Post-Vaccine	*p*
*n*	13	20	**0.002**
Gender (male %)	6 (46.15)	6 (30)	0.346
Age (years)	44.23 ± 20.77	49.50 ± 23.25	0.512
Albumin (g/dL)	2.2 (2.05–3.30)	2.3 (1.93–3.45)	0.825
Creatinine (mg/dL)	0.65 (0.55–1.74)	0.88 (0.67–1.01)	0.302
Proteinuria (g/g)	8.67 (1.67–12.31)	4.85 (2.50–10.79)	0.478
Remission < 1 month (%)	2 (15.38%)	7 (35%)	**0.005**
Remission 1–3 months (%)	8 (61.54%)	8 (40%)	**0.02**
Remission 3–6 months (%)	1 (7.69%)	3 (15%)	**0.02**
No remission 6–12 months (%)	0 (0%)	2 (10%)	0.508

Data are expressed as *n* (%), mean ± SD or median (interquartile range—IQR-: 25th–75th percentiles). *p* values less than 0.05 are considered statistically significant (bold values). MCD: minimal change disease.

**Table 5:** Demographic and laboratory characteristics of patients with IMG <6 weeks after SARS-CoV-2 individual vaccination.

IMG (%)	17 (100)
MCD (%)	1 (5.9)
FSGS (%)	1 (5.9)
IgAN (%)	5 (29.4)
Vasculitis (%)	2 (11.8)
Anti-GBM disease (%)	2 (11.8)
Lupus nephritis (%)	3 (17.5)
IgG4 disease (%)	1 (5.9)
MPGN (%)	1 (5.9)
Gender (male %)	8 (47.10)
Age (years)	50.47 ± 15.88
Albumin (g/dL)	3.5 (3.05–4.30)
Creatinine (mg/dL)	1.75 (0.77–5.22)
Proteinuria (g/g)	2.41 (0.82–6.06)
Hematuria (yes %)	9 (52.9)
mRNA vaccine (%)	14 (82.4)
Flare after 1st vaccine dose (%)	9 (52.9)
Flare after 2nd vaccine dose (%)	4 (23.5)
Flare after 3rd vaccine dose (%)	4 (23.5)

Data are expressed as *n* (%), mean ± SD or median (interquartile range—IQR-: 25th–75th percentiles). IMG: immune-mediated glomerulopathies. MCD: minimal change disease. FSGS: focal and segmental glomerulosclerosis. IgAN: immunoglobulin A nephropathy. Anti-GBM disease: anti-glomerular basement membrane disease. IgG4 disease: immunoglobulin G4-related disease. MPGN: membranoproliferative glomerulonephritis. mRNA vaccine: messenger ribonucleic acid vaccine.

## Data Availability

Data are available upon reasonable request.
